# Supplementary figures and images for: Development of a conceptual framework for defining trial efficiency
Source: PLoS One. 2024 May 23;19(5):e0304187. doi: 10.1371/journal.pone.0304187 (PMC11115328; doi:10.1371/journal.pone.0304187)

**S2 Fig. Trial Process in General**


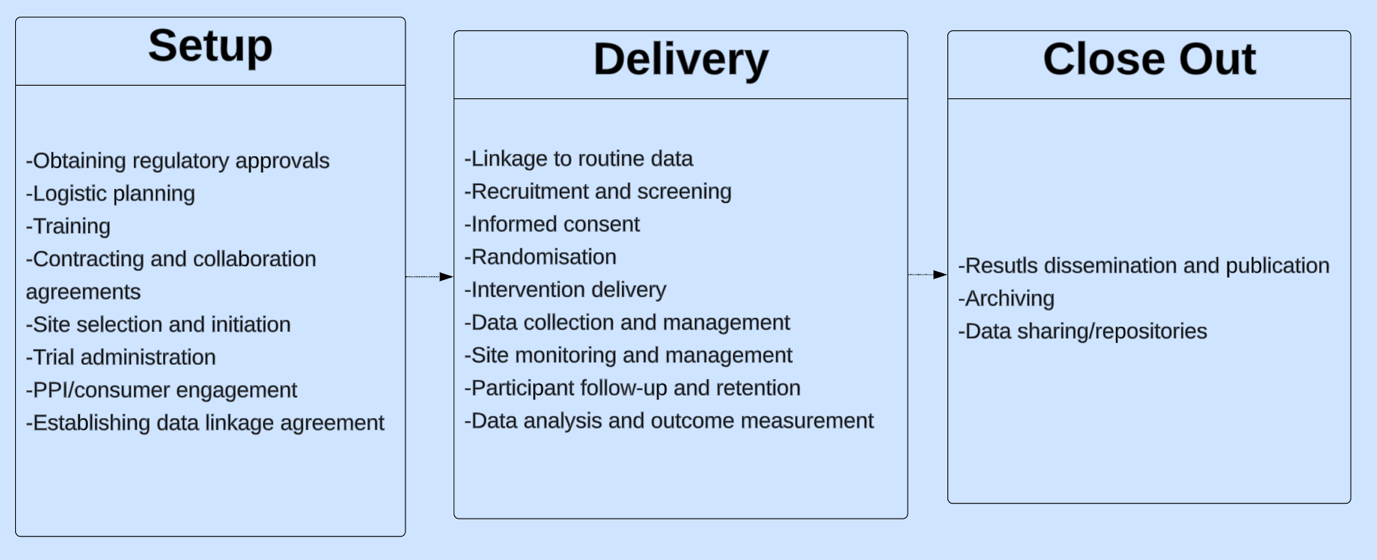

Supplement: S2 Fig — (DOCX) [file pone.0304187.s002.docx]
