# Supplementary material for: Development of a conceptual framework for defining trial efficiency
Source: PLoS One. 2024 May 23;19(5):e0304187. doi: 10.1371/journal.pone.0304187 (PMC11115328; doi:10.1371/journal.pone.0304187)
Supplement: S1 Table — (DOCX) [file pone.0304187.s003.docx]

**S1 Table. Literature Review Inclusion and Exclusion Criteria**

| **Inclusion** | **Exclusion** |
| --- | --- |
| Any type of trial design | Non-English studies |
| Any type of studies | Studies did not discuss efficiency in terms of trial aspects |
| Studies used the term ‘(in)efficien*’ in the titles or keywords | Studies focused on the efficiency of interventions |

Footnote: Table was derived from the literature review preprint : Xie CX. How have researchers defined and used the concept of ‘efficiency’ in the context of trials? A review of existing literature and a proposed conceptual framework [Internet]. OSF Preprints. 2023. Available from: osf.io/tms89.
