# Supplementary material for: Development of a conceptual framework for defining trial efficiency
Source: PLoS One. 2024 May 23;19(5):e0304187. doi: 10.1371/journal.pone.0304187 (PMC11115328; doi:10.1371/journal.pone.0304187)
Supplement: S2 Table — (DOCX) [file pone.0304187.s004.docx]

**S2 Table. Efficiency Definitions/explanations in the Literature**

| **Studies** | **What is meant by efficiency?** |
| --- | --- |
| Howat and Holloway 1977^1^ | “The term efficiency is used to mean the relative cost of achieving a specific objective.” |
| Friedewald 1990^2^ | “Efficiency has been defined as ‘the capacity to produce desired results with a minimum expenditure of energy, time, money, or materials.” |
| Mdege et al., 2014^3^ | “The most commonly cited reason for the factorial design was efﬁciency (ie, to assess the effects of two interventions independently in the same study)”. |
| Lauer et al., 2017^4^ | “The GISSI trials were efficient, in part, because the trials were well integrated into routine clinical care.” |
| Cornelius et al., 2018^5^ | “The notion of ‘efficiency’ encompasses a broad range of methodological approaches, including innovative designs, logistical planning, and novel approaches to recruitment and outcome data collection, which may be employed to reduce the level of resources required to set up and conduct a trial or to enhance the value of trial investments by enabling longer-term follow-up in usual care settings.” |
| Brunet et al., 2019^6^ | “Efficiency refers to the ability to achieve a therapeutic goal using a lesser amount of time and resources than what is typically observed.” |
| Brown et al.,2022^7^ | “The efficiency is that more patients are being used for multiple sub-trials rather than just selecting patients with one particular biomarker and excluding the rest.” |
| He et al., 2022^8^ | “Efﬁciency is a measure of the utility of a study per unit of resource (number of trial participants and/or ﬁnancial cost) expended in the study or as a consequence of the study.” |

Footnote: Table was derived from the literature review preprint : Xie CX. How have researchers defined and used the concept of ‘efficiency’ in the context of trials? A review of existing literature and a proposed conceptual framework [Internet]. OSF Preprints. 2023. Available from: osf.io/tms89.

**Table 2 References:**

1. Howat AP, Holloway PJ. The effect of diagnostic criteria on the efficiency of experimental clinical trials. *J Dent Res* 1977;56 Spec No:C116-22. doi: 10.1177/002203457705600303011
2. Friedewald WT. Costs of clinical trials and the need for efficiency: a brief overview. *Stat Med* 1990;9(1-2):9-12. doi: 10.1002/sim.4780090106
3. Mdege ND, Brabyn S, Hewitt C, et al. The 2 x 2 cluster randomized controlled factorial trial design is mainly used for efficiency and to explore intervention interactions: a systematic review. *Journal of Clinical Epidemiology* 2014;67(10):1083-92.
4. Lauer MS, Gordon D, Wei G, et al. Efficient design of clinical trials and epidemiological research: is it possible? *Nat Rev Cardiol* 2017;14(8):493-501. doi: 10.1038/nrcardio.2017.60 [published Online First: 20170427]
5. Cornelius VR, McDermott L, Forster AS, et al. Automated recruitment and randomisation for an efficient randomised controlled trial in primary care. *Trials* 2018;19(1):341. doi: 10.1186/s13063-018-2723-3
6. Brunet A, Ayrolles A, Gambotti L, et al. Paris MEM: a study protocol for an effectiveness and efficiency trial on the treatment of traumatic stress in France after the 2015-16 terrorist attacks. *BMC Psychiatry* 2019;19(1):351.
7. Brown LC, Graham J, Fisher D, et al. Experiences of running a stratified medicine adaptive platform trial: Challenges and lessons learned from 10 years of the FOCUS4 trial in metastatic colorectal cancer. *Clin Trials* 2022;19(2):146-57. doi: 10.1177/17407745211069879 [published Online First: 20220127]
8. He L, Ren Y, Chen H, et al. Efficiency of a randomized confirmatory basket trial design constrained to control the family wise error rate by indication. *Stat Methods Med Res* 2022;31(7):1207-23. doi: 10.1177/09622802221091901 [published Online First: 20220411]
