# Supplementary material for: Development of a conceptual framework for defining trial efficiency
Source: PLoS One. 2024 May 23;19(5):e0304187. doi: 10.1371/journal.pone.0304187 (PMC11115328; doi:10.1371/journal.pone.0304187)
Supplement: S3 Table — (DOCX) [file pone.0304187.s005.docx]

**S3 Table. Scoring Round Stratified Results**

Table 3.1: Appropriateness, disagreement, median item ratings, inter-percentile range, and inter-percentile range adjusted for asymmetry - Funders and Sponsors

| Item | Appropriateness | Disagreement | Median | P_30_ | P_70_ | IPR | IPRAS |
| --- | --- | --- | --- | --- | --- | --- | --- |
| 1.1 | Appropriate | No | 9 | 8 | 9 | 1 | 7.6 |
| 1.2 | Appropriate | No | 9 | 8 | 9 | 1 | 7.6 |
| 1.3 | Appropriate | No | 8 | 7 | 9 | 2 | 6.85 |
| 1.4 | Appropriate | No | 8 | 7 | 9 | 2 | 6.85 |
| 2.1 | Appropriate | No | 8.5 | 8 | 9 | 1 | 7.6 |
| 2.2 | Appropriate | No | 8.5 | 8 | 9 | 1 | 7.6 |
| 2.3 | Appropriate | No | 7.5 | 7 | 8 | 1 | 6.1 |
| 2.4 | Appropriate | No | 8 | 8 | 8 | 0 | 6.85 |
| 2.5 | Appropriate | Yes | 7.5 | 3 | 8 | 5 | 3.1 |

1.1 Scientiﬁc efﬁciency: methodological rigour of the trial design

1.2 Operational efﬁciency: optimal management, organization, and execution of trial processes and procedures

1.3 Statistical efﬁciency: a measure of quality of an estimator, of an experimental design, or of a hypothesis testing procedure

1.4 Economic efﬁciency: optimal use of resources in the design, implementation, and analysis of clinical trials

2.1 Trial design: planning and organisation of a trial

2.2 Trial process: trial set up, conduct and close out

2.3 Stakeholders: individuals or groups who have an interest or concern in the design, execution, and outcomes of a trial

2.4 Infrastructure: underlying framework, systems, and resources required to design, implement, manage, and analyse a trial

2.5 Superstructure: overarching structure of a trial

IPR: inter-percentile range

IPRAS: inter-percentile range adjusted for symmetry

Table 3.2: Appropriateness, disagreement, median item ratings, inter-percentile range, and inter-percentile range adjusted for asymmetry – Statisticians

| Item | Appropriateness | Disagreement | Median | P_30_ | P_70_ | IPR | IPRAS |
| --- | --- | --- | --- | --- | --- | --- | --- |

| 1.1 | Appropriate | No | 9 | 8 | 9 | 1 | 7.6 |
| --- | --- | --- | --- | --- | --- | --- | --- |
| 1.2 | Appropriate | No | 8 | 8 | 9 | 1 | 7.6 |
| 1.3 | Appropriate | No | 8 | 7 | 9 | 2 | 6.85 |
| 1.4 | Appropriate | No | 8 | 6 | 8 | 2 | 5.35 |
| 2.1 | Appropriate | No | 9 | 9 | 9 | 0 | 8.35 |
| 2.2 | Appropriate | No | 8 | 8 | 9 | 1 | 7.6 |
| 2.3 | Appropriate | No | 7 | 7 | 9 | 2 | 6.85 |
| 2.4 | Appropriate | No | 8 | 8 | 9 | 1 | 7.6 |
| 2.5 | Appropriate | No | 8 | 6 | 9 | 3 | 6.1 |

1.1 Scientiﬁc efﬁciency: methodological rigour of the trial design

1.2 Operational efﬁciency: optimal management, organization, and execution of trial processes and procedures

1.3 Statistical efﬁciency: a measure of quality of an estimator, of an experimental design, or of a hypothesis testing procedure

1.4 Economic efﬁciency: optimal use of resources in the design, implementation, and analysis of clinical trials

2.1 Trial design: planning and organisation of a trial

2.2 Trial process: trial set up, conduct and close out

2.3 Stakeholders: individuals or groups who have an interest or concern in the design, execution, and outcomes of a trial

2.4 Infrastructure: underlying framework, systems, and resources required to design, implement, manage, and analyse a trial

2.5 Superstructure: overarching structure of a trial

IPR: inter-percentile range

IPRAS: inter-percentile range adjusted for symmetry

Table 3.3: Appropriateness, disagreement, median item ratings, inter-percentile range, and inter-percentile range adjusted for asymmetry - Trial Managers

| Item | Appropriateness | Disagreement | Median | P_30_ | P_70_ | IPR | IPRAS |
| --- | --- | --- | --- | --- | --- | --- | --- |

| 1.1 | Appropriate | No | 8.5 | 8 | 9 | 1 | 7.6 |
| --- | --- | --- | --- | --- | --- | --- | --- |
| 1.2 | Appropriate | No | 8.5 | 8 | 9 | 1 | 7.6 |
| 1.3 | Appropriate | No | 8 | 8 | 8.5 | .5 | 7.225 |
| 1.4 | Appropriate | No | 8 | 8 | 8 | 0 | 6.85 |
| 2.1 | Appropriate | No | 9 | 8 | 9 | 1 | 7.6 |
| 2.2 | Appropriate | No | 9 | 8 | 9 | 1 | 7.6 |
| 2.3 | Appropriate | No | 8.5 | 8 | 9 | 1 | 7.6 |
| 2.4 | Appropriate | No | 8 | 7.5 | 9 | 1.5 | 7.225 |
| 2.5 | Appropriate | No | 8 | 6.5 | 8 | 1.5 | 5.725 |

1.1 Scientiﬁc efﬁciency: methodological rigour of the trial design

1.2 Operational efﬁciency: optimal management, organization, and execution of trial processes and procedures

1.3 Statistical efﬁciency: a measure of quality of an estimator, of an experimental design, or of a hypothesis testing procedure

1.4 Economic efﬁciency: optimal use of resources in the design, implementation, and analysis of clinical trials

2.1 Trial design: planning and organisation of a trial

2.2 Trial process: trial set up, conduct and close out

2.3 Stakeholders: individuals or groups who have an interest or concern in the design, execution, and outcomes of a trial

2.4 Infrastructure: underlying framework, systems, and resources required to design, implement, manage, and analyse a trial

2.5 Superstructure: overarching structure of a trial

IPR: inter-percentile range

IPRAS: inter-percentile range adjusted for symmetry

Table 3.4: Appropriateness, disagreement, median item ratings, inter-percentile range, and inter-percentile range adjusted for asymmetry – Principal Investigators

| Item | Appropriateness | Disagreement | Median | P_30_ | P_70_ | IPR | IPRAS |
| --- | --- | --- | --- | --- | --- | --- | --- |

| 1.1 | Appropriate | No | 8.5 | 7 | 9 | 2 | 6.85 |
| --- | --- | --- | --- | --- | --- | --- | --- |
| 1.2 | Appropriate | No | 8.5 | 8 | 9 | 1 | 7.6 |
| 1.3 | Appropriate | No | 8 | 7 | 9 | 2 | 6.85 |
| 1.4 | Appropriate | No | 7.5 | 6 | 8 | 2 | 5.35 |
| 2.1 | Appropriate | No | 9 | 8 | 9 | 1 | 7.6 |
| 2.2 | Appropriate | No | 8 | 8 | 9 | 1 | 7.6 |
| 2.3 | Appropriate | No | 8 | 7 | 9 | 2 | 6.85 |
| 2.4 | Appropriate | No | 9 | 9 | 9 | 0 | 8.35 |
| 2.5 | Appropriate | No | 8.5 | 8 | 9 | 1 | 7.6 |

1.1 Scientiﬁc efﬁciency: methodological rigour of the trial design

1.2 Operational efﬁciency: optimal management, organization, and execution of trial processes and procedures

1.3 Statistical efﬁciency: a measure of quality of an estimator, of an experimental design, or of a hypothesis testing procedure

1.4 Economic efﬁciency: optimal use of resources in the design, implementation, and analysis of clinical trials

2.1 Trial design: planning and organisation of a trial

2.2 Trial process: trial set up, conduct and close out

2.3 Stakeholders: individuals or groups who have an interest or concern in the design, execution, and outcomes of a trial

2.4 Infrastructure: underlying framework, systems, and resources required to design, implement, manage, and analyse a trial

2.5 Superstructure: overarching structure of a trial

IPR: inter-percentile range

IPRAS: inter-percentile range adjusted for symmetry

Table 3.5: Appropriateness, disagreement, median item ratings, inter-percentile range, and inter-percentile range adjusted for asymmetry - PPIs

| Item | Appropriateness | Disagreement | Median | P_30_ | P_70_ | IPR | IPRAS |
| --- | --- | --- | --- | --- | --- | --- | --- |

| 1.1 | Appropriate | No | 9 | 6 | 9 | 3 | 6.1 |
| --- | --- | --- | --- | --- | --- | --- | --- |
| 1.2 | Appropriate | No | 8 | 5 | 9 | 4 | 5.35 |
| 1.3 | Appropriate | No | 9 | 6 | 9 | 3 | 6.1 |
| 1.4 | Appropriate | No | 8 | 6 | 9 | 3 | 6.1 |
| 2.1 | Appropriate | No | 9 | 8 | 9 | 1 | 7.6 |
| 2.2 | Appropriate | No | 9 | 9 | 9 | 0 | 8.35 |
| 2.3 | Appropriate | No | 9 | 7 | 9 | 2 | 6.85 |
| 2.4 | Appropriate | No | 7 | 7 | 9 | 2 | 6.85 |
| 2.5 | Appropriate | No | 7 | 7 | 9 | 2 | 6.85 |

1.1 Scientiﬁc efﬁciency: methodological rigour of the trial design

1.2 Operational efﬁciency: optimal management, organization, and execution of trial processes and procedures

1.3 Statistical efﬁciency: a measure of quality of an estimator, of an experimental design, or of a hypothesis testing procedure

1.4 Economic efﬁciency: optimal use of resources in the design, implementation, and analysis of clinical trials

2.1 Trial design: planning and organisation of a trial

2.2 Trial process: trial set up, conduct and close out

2.3 Stakeholders: individuals or groups who have an interest or concern in the design, execution, and outcomes of a trial

2.4 Infrastructure: underlying framework, systems, and resources required to design, implement, manage, and analyse a trial

2.5 Superstructure: overarching structure of a trial

IPR: inter-percentile range

IPRAS: inter-percentile range adjusted for symmetry
