# Supplementary material for: Development of a conceptual framework for defining trial efficiency
Source: PLoS One. 2024 May 23;19(5):e0304187. doi: 10.1371/journal.pone.0304187 (PMC11115328; doi:10.1371/journal.pone.0304187)
Supplement: S4 Table — (DOCX) [file pone.0304187.s006.docx]

**S4 Table. Scoring Round Exemplar Quotes Related to Potential Overlapping Constructs**

| Overlap between statistical efficiency and scientific efficiency | Quote 1: “I do not really understand how scientific efficiency is distinct from statistical efficiency.” (Participant n.8, statistician) |
| --- | --- |
|  | Quote 2: “This (scientific efficiency) may encompass statistical efficiency as well. I’m not sure why it wouldn’t nor why it would be distinct.” (Participant n.11, funder) |
|  | Quote 3: “This (statistical efficiency) seems to overlap with 1.1 scientific efficiency. It is part of the methodology.” (Participant n.9, member of the public) |
|  |  |
| Overlap between economic efficiency and operational efficiency | Quote 4: “This (operational efficiency) might include economic efficiency. It would be helpful to tease out the distinction between this and economic efficiency.” (Participant n.11, funder) |
|  | Quote 5: “I'm not sure I have a better idea but aspects of this construct (economic efficiency) seem to overlap with other constructs, particularly operational efficiency, which by definition would be economically efficient.” (Participant n.7, ﻿journal editor) |
|  | Quote 6: “For me this (economic efficiency) is just the same as operational efficiency.” (Participant n.1, statistician) |
