# Supplementary material for: Development of a conceptual framework for defining trial efficiency
Source: PLoS One. 2024 May 23;19(5):e0304187. doi: 10.1371/journal.pone.0304187 (PMC11115328; doi:10.1371/journal.pone.0304187)
Supplement: S1 File — (DOCX) [file pone.0304187.s007.docx]

**S1 File. Open Round Questionnaire**

**What do you think about the EFFICIENCY within the context of randomised trials?**

# Introduction

This e-Delphi study aims to increase our understanding of efficiency within the context of randomised trials in health service and implementation research.

Your participation in this study and your individual responses will be strictly confidential to the research team and will not be disclosed to any outside party, including other participants. Please contact us with any questions or concerns you may have regarding the study.

# Why are we asking you to take part?

You are invited to consider taking part in this study because we believe you are familiar with randomised trials and have experience working on or participating in trial conduct, management, oversight, methodology and funding.

# What are we asking you to do?


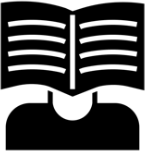


Study stage 1 completed. We reviewed the existing literature and collated how researchers have defined and used the concept of ‘efficiency’ in the context of trials.


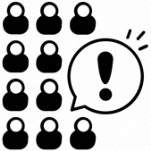


**Study stage 2** – Invitation to join online Delphi study. We will email you a link to start the first round.

*We will ask your opinions on trial efficiency. Up to 6 weeks to complete. The final date you can complete R1 is 24 th March at 23:59 GMT.*


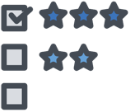

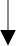


We will email you a link to take part in the second round.

*We will show you the synthesised R1 results, combined with literature review findings. You will rate the level of importance accordingly. You can suggest additional items. R2 will be available around 2 weeks after the end of R1. Up to 3 weeks to complete.*


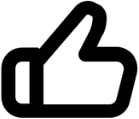


We will email you a link to take part in the third round.

*We will show you the results from R2, you will see what others have rated important. You can change your ratings and make final decisions. R3 will be available around 2 weeks after the end of R2. Up to 3 weeks to complete.*


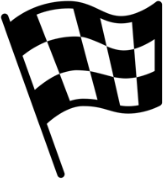


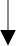


End of study

*We will publish the results and the plan for future research.*

# Privacy notice

This privacy notice describes how we collect and use your personal data in accordance with the UK General Data Protection Regulation (UK-GDPR). The legal basis under which we process personal data is "public task", where processing is necessary for the performance of a task carried out in the public interest or in the exercise of official authority vested in the controller.

Queen Mary University of London (QMUL) is the "data controller" for the information that will be collected. Clinvivo is the "data processor" and will process personal data on behalf of the controller. Clinvivo's privacy policy can be found here: [<http://www.clinvivo.com/privacy.html>]. For this study, Clinvivo note that a slight variation on the security of data storage is that they store a cryptographic key in the link they send you for the Delphi questionnaires. This is so that in subsequent Delphi questionnaires Clinvivo can present to you the choice you made previously but still keep that information encrypted on their server.

# The type of personal information to be collected

We will collect and process the following information about you such as your: name, email, ratings and views about trial efficiency, current roles within a trial, country of residence.

# How we get the personal information and why we have it

Most of the personal information we process is provided to us directly by you, except for your email address that was within the public domain. The results from this research and the resulting trial efficiency framework will be published in peer-reviewed journals, conferences and Charis’ thesis in anonymised format. Please refer to the QMUL Privacy Notice for Research Participants for further details, including your rights, which can be found here.

# How we store your personal information

Clinvivo as data processors will store data for up to 12 months after the completion of the final Delphi study after which all electronic information will be permanently deleted. Your data will not be shared with third parties, will be processed only for the purpose of the study, and will be stored in anonymous

format. Your data will be stored on Clinvivo’s secure server and securely transferred to the QMUL

secure data safe haven from where it will only be accessible to the research team.

Your data will be kept for five years after the research study has finished, defined as once the study has been published, in line with QMUL’s Records Retention Schedule and then deleted following QMUL’s disposal of information policy, whereby the data is overwritten many times so it cannot be recovered.

# Our contact details

If you have any questions about the study, please contact Charis Xie [<cha](mailto:charis.xie@qmul.ac.uk)r[is.xie@qmul.ac.uk>.](mailto:charis.xie@qmul.ac.uk)

**1: Basic information**

- 1. **Name**
  2. **Email**
  3. **Country of residence**
  4. **What best describes your current roles in a trial? (Please select all that apply)**
     - Funder
     - Sponsor
     - Principal trial investigator
     - Trial manager
     - Statistician
     - Data manager
     - Trial participant
     - Site staff
     - Member of the public/PPI
     - Journal editor
     - Other

# : How do you define efficiency within the context of trials to improve healthcare?

*[You may suggest more than one definition; you may use/combine part of the existing definitions reported below, if so, please tailor it to your own understanding]*

*[Please put any comment you may have that relates to your specific wording and terms]*

**Here are some definitions from dictionaries and literature. Some definitions from dictionaries:**

- - - The ratio of the useful work done by a machine, engine, device, etc, to the energy supplied to it, often expressed as a percentage
    - The production of the desired effects or results with minimum waste of time, effort, or skill.
    - The ratio of the effective or useful output to the total input in any system.

# Some definitions from clinical trial literature:

- - - “Efficiency has been defined as the capacity to produce desired results with a minimum expenditure of energy, time, money, or materials.” (Friedewald, 1990)
    - “Efﬁciency is a measure of the utility of a study per unit of resource (number of trial participants and/or ﬁnancial cost) expended in the study or as a consequence of the study.” (He et al., 2022)
    - “The term efficiency is used to mean the relative cost of achieving a specific

objective.”( Howat and Holloway, 1977)

- - - “The notion of ‘efficiency’ encompasses a broad range of methodological approaches, including innovative designs, logistical planning, and novel approaches to recruitment and outcome data collection, which may be employed to reduce the level of resources required to set up and conduct a trial or to enhance the value of trial investments by enabling longer-term follow-up in usual care settings.” (Cornelius et al., 2018)
    - “Efficiency refers to the ability to achieve a therapeutic goal using a lesser amount of

time and resources than what is typically observed.” (Brunet et al., 2019)

- 1. **: Is there any other comment on your definition?**

# What would you consider to have been the most efficient OR inefficient aspects in the trials you have conducted/participated in? Please specify at least one aspect:

**Here are some examples from the literature review we conducted:**

- - Innovative trial designs (e.g. adaptive designs, cluster designs, basket trials, platform trials, Trials within Cohorts design)
  - Increased operational efficiency in terms of patient identification and recruitment, data collection and management, data analysis, site selection and management.
  - Other: applying information technologies and use of routine healthcare data; involving the public and stakeholders; efficient trial reviews and regulatory approvals.

# 4: Do you have any other comments?

Thank you, please click Submit to finish the 1^st^ round of this study.

Submit
